# Effects of exercise adherence on subjective wellbeing in middle-aged and older adults: a three-wave longitudinal chain mediation study

**DOI:** 10.3389/fpsyg.2026.1923252

**Published:** 2026-08-27

**Authors:** Jixin Wang, Hui Jia, Hui Wang

**Affiliations:** 1 College of Physical Education, Changzhou University, Changzhou, Jiangsu, China; 2 Division of Marine Sports, Pukyong National University, Busan, Republic of Korea; 3 School of Physical Education, Zhengzhou University, Zhengzhou, Henan, China; 4 School of Nursing, Shandong First Medical University & Shandong Academy of Medical Sciences, Tai’an, Shandong, China

**Keywords:** exercise adherence, exercise identity, longitudinal mediation, middle-aged and older adults, positive emotional expressivity, subjective wellbeing

## Abstract

**Objective:**

This study examined whether exercise identity and positive emotional expressivity statistically explain the prospective association between exercise adherence and subjective wellbeing among middle-aged and older adults. Drawing on identity theory, self-determination perspectives, and the broaden-and-build theory of positive emotions, we tested a three-wave longitudinal chain mediation model while controlling for baseline subjective wellbeing and demographic covariates.

**Methods:**

Community-dwelling adults aged 45–75 years were recruited from community centers, senior universities, and public health platforms in Zhejiang Province, China. At baseline, 550 eligible participants completed measures of exercise adherence, subjective wellbeing, and covariates. Exercise identity was assessed 6 months later, and positive emotional expressivity and subjective wellbeing were assessed at 12 months. The final completer sample included 411 participants (mean age = 58.72 years, SD = 6.81; age range = 45–75 years; 180 men [43.8%] and 231 women [56.2%]). Attrition analyses, confirmatory factor analysis, longitudinal measurement invariance of subjective wellbeing, and structural equation modeling with 5,000 bootstrap samples were conducted.

**Results:**

Attrition analyses indicated no significant baseline differences between retained and attrited participants. The measurement model showed acceptable reliability, convergent validity, and discriminant validity. The structural model fit the data well (*χ*^2^/df = 1.89, CFI = 0.967, TLI = 0.960, RMSEA = 0.045, SRMR = 0.041). Exercise adherence at baseline was positively associated with subjective wellbeing at 12 months in the total-effect model (*β* = 0.240, *p* < 0.001). In the mediation model, the direct path was attenuated and became non-significant (*β* = 0.079, *p* = 0.126). The indirect paths through exercise identity (*β* = 0.068), positive emotional expressivity (*β* = 0.036), and the sequential path through exercise identity and positive emotional expressivity (*β* = 0.057, 95% CI [0.028, 0.094]) were significant.

**Conclusion:**

Findings suggest that sustained exercise adherence may be linked to later subjective wellbeing partly through the consolidation of exercise identity and the expression of positive emotions. Because the final mediator and outcome were assessed at the same wave and the design was observational, causal claims should be interpreted cautiously. Community exercise programs for aging populations may benefit from strategies that support exercise-related self-concept and positive affective expression in addition to promoting physical activity itself.

## 1 Introduction

Population aging has become a central public health and social policy issue. The World Health Organization (2020) has emphasized that physical activity is relevant to adults and older adults not only for disease prevention but also for functional capacity and healthy aging. China is experiencing an especially marked aging transition: the Seventh National Population Census reported that adults aged 60 years and older accounted for 18.70% of the national population in 2020, a substantial increase from 2010 (National Bureau of Statistics of China, 2021). In this context, subjective wellbeing is an important indicator of successful aging because it reflects how individuals cognitively and affectively evaluate their lives and is closely associated with health, functioning, and longevity in later life (Diener et al., 2018; Steptoe et al., 2015). Although successful aging has often been discussed in terms of physical function and disease avoidance, psychological adaptation and perceived life quality are also central to aging well (Ryff, 1989).

Regular physical activity is widely recognized as a modifiable behavior associated with physical health, functional independence, quality of life, and psychological wellbeing in later adulthood. Seminal reviews and intervention meta-analyses have generally shown that physical activity is positively related to quality of life and wellbeing-related outcomes in older adults (Biddle et al., 2000; Netz et al., 2005; Rejeski and Mihalko, 2001). More recent evidence also indicates a positive association between physical activity and subjective wellbeing in healthy populations, although effect sizes vary across measures, samples, and study designs (Buecker et al., 2021). Clinical and public health guidelines therefore encourage older adults to engage in regular aerobic, muscle-strengthening, balance, and multicomponent activities within their capacity (Bull et al., 2020; Chodzko-Zajko et al., 2009).

However, the translation of physical activity recommendations into sustained behavior remains challenging. Many adults intend to be active but fail to maintain regular activity over time, a pattern consistent with the well-documented intention-behavior gap in physical activity research (Rhodes and de Bruijn, 2013). Behavior-maintenance theories further suggest that long-term adherence requires self-regulation, habit formation, perceived reward, identity integration, and supportive social contexts rather than simple knowledge of health benefits alone (Kwasnicka et al., 2016). For middle-aged and older adults, exercise adherence is therefore a more psychologically meaningful construct than isolated exercise frequency because it captures whether exercise has become stable, deliberate, and resilient to everyday barriers such as fatigue, competing family responsibilities, weather conditions, or minor discomfort.

One theoretical pathway linking adherence to wellbeing is exercise identity. Identity theory proposes that repeated role-consistent behaviors can strengthen the salience of corresponding role identities within the self-concept (Stryker, 1968; Stryker and Burke, 2000). In the exercise domain, exercise identity refers to the extent to which individuals see being an exerciser as part of who they are, and it has been operationalized with established scales assessing the salience of exercise in self-definition (Anderson and Cychosz, 1994). Meta-analytic evidence suggests that physical activity identity and related self-schema constructs are reliably associated with physical activity behavior (Rhodes et al., 2016). Recent evidence from Türkiye further showed that exercise identity was positively associated with mental wellbeing, with self-liking and self-competence serving as significant mediators (Demir Kaya and Kaya, 2026). Related research among Turkish university students also found that self-esteem mediated the association between perceived parenting styles and subjective wellbeing, providing broader evidence for the role of self-evaluative processes in wellbeing (Odacı et al., 2026). For adults navigating role transitions related to retirement, family changes, and aging-related health concerns, sustained exercise may provide a salient behavioral basis for developing a health-oriented self-concept. This identity may then help transform exercise from an externally recommended activity into a personally meaningful role, a process also consistent with self-determination theory’s emphasis on internalization and autonomous motivation (Deci and Ryan, 2000).

A second pathway may involve positive emotional expressivity. The broaden-and-build theory of positive emotions argues that positive emotions broaden thought-action repertoires and, over time, build enduring psychological and social resources (Fredrickson, 2001, 2004). In health behavior contexts, positive affective processes have been theorized to support upward spirals of behavior maintenance by increasing reward value, implicit motivation, and openness to future engagement (van Cappellen et al., 2018). Emotional expressivity represents the tendency to outwardly display affective states through facial, verbal, and bodily channels (Gross and John, 1997). When exercise becomes part of one’s identity, positive feelings experienced during exercise may be more readily expressed and shared. Such expression may reinforce social connection, accomplishment, and positive affective meaning in exercise settings, thereby contributing to broader wellbeing.

The affective mechanism may be particularly relevant in later adulthood. Socioemotional selectivity theory argues that as perceived time horizons become more limited, people increasingly prioritize emotionally meaningful goals and relationships (Carstensen et al., 1999). Reviews of emotional aging likewise show that older adults often place high value on affect regulation, emotionally meaningful interactions, and stability of positive social ties (Charles and Carstensen, 2010). Community exercise environments may therefore serve not only as settings for physical exertion but also as structured contexts in which older adults experience mastery, mutual encouragement, and shared positive emotions. This makes positive emotional expressivity a plausible proximal psychological process linking exercise-related identity to broader subjective wellbeing. Accordingly, evaluations of community exercise programs may consider not only attendance, intensity, and physical function but also exercise identity and positive emotional expressivity, particularly because exercise participation among middle-aged and older adults may be shaped by health status, family obligations, social support, and access to exercise resources (Steptoe et al., 2015).

**Figure 1:**
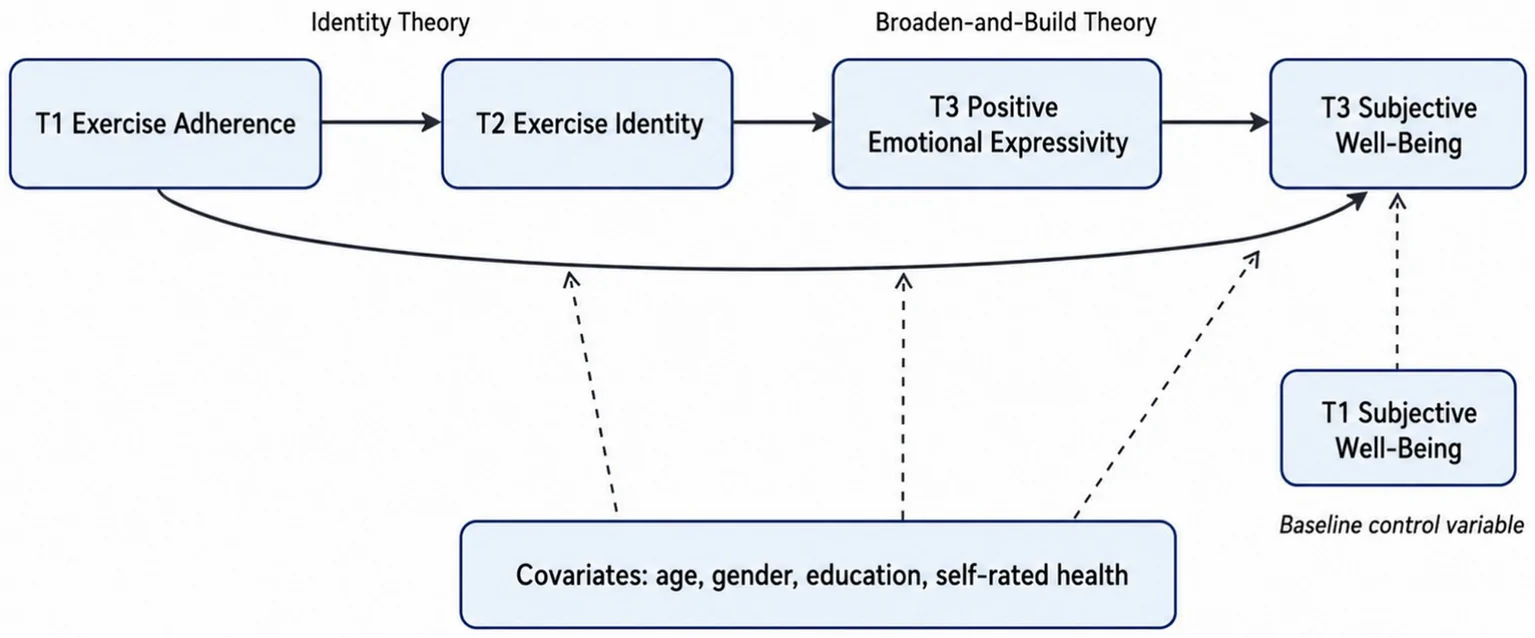
Conceptual model of the proposed longitudinal mediation pathway.

Building on these perspectives, the present study tested a three-wave longitudinal mediation model (Figure 1) in which baseline exercise adherence predicted exercise identity 6 months later, which in turn predicted positive emotional expressivity and subjective wellbeing at 12 months. The design improves upon purely cross-sectional mediation models by establishing temporal ordering between the predictor and first mediator and by controlling for baseline subjective wellbeing. At the same time, because positive emotional expressivity and follow-up subjective wellbeing were measured at the same final wave, the proposed chain mediation should be interpreted as a prospective model with a concurrent final-wave mediator-outcome association rather than definitive causal evidence. This cautious interpretation is important because longitudinal structural models can strengthen temporal inference but do not, by themselves, eliminate unmeasured confounding or prove causal mechanisms (Little, 2013).

We tested four hypotheses. H1: Baseline exercise adherence would be positively associated with 12-month subjective wellbeing after controlling for baseline subjective wellbeing and covariates. H2: Exercise identity at 6 months would statistically mediate the association between baseline exercise adherence and 12-month subjective wellbeing. H3: Positive emotional expressivity at 12 months would statistically mediate the association between baseline exercise adherence and 12-month subjective wellbeing. H4: Exercise identity and positive emotional expressivity would form a significant sequential indirect pathway linking exercise adherence to later subjective wellbeing.

## 2 Methods

### 2.1 Participants and procedure

Participants were recruited from 12 urban community centers, four senior universities, and three local public health digital platforms in Zhejiang Province, China. Recruitment combined convenience sampling and targeted community outreach with assistance from community health workers. Inclusion criteria were as follows: age between 45 and 75 years; no physician-diagnosed severe cognitive impairment; ability to complete questionnaires independently; no severe motor limitation or medical contraindication that would prevent regular exercise; and willingness to participate in a 12-month longitudinal survey. Participants were informed that the study focused on exercise behavior and psychological adaptation in adulthood and later life, that participation was voluntary, and that they could withdraw at any time. All participants provided written informed consent before participation. To assess the adequacy of our sample size for structural equation modeling, a post-hoc sensitivity power analysis was conducted using the semTools package in R, based on the framework developed by MacCallum et al. (1996). For our final structural model with 212 degrees of freedom, the retained sample size of 411 provided a statistical power of > 0.99 to reject a not-close fit (RMSEA = 0.08) in favor of a close fit (RMSEA = 0.05) at an alpha level of 0.05. This calculation indicated that the retained sample provided high power for the RMSEA-based test of global model fit. Because this post-hoc calculation does not directly evaluate power for individual structural or indirect effects, it was treated as supplementary evidence of sample-size adequacy.

**Figure 2:**
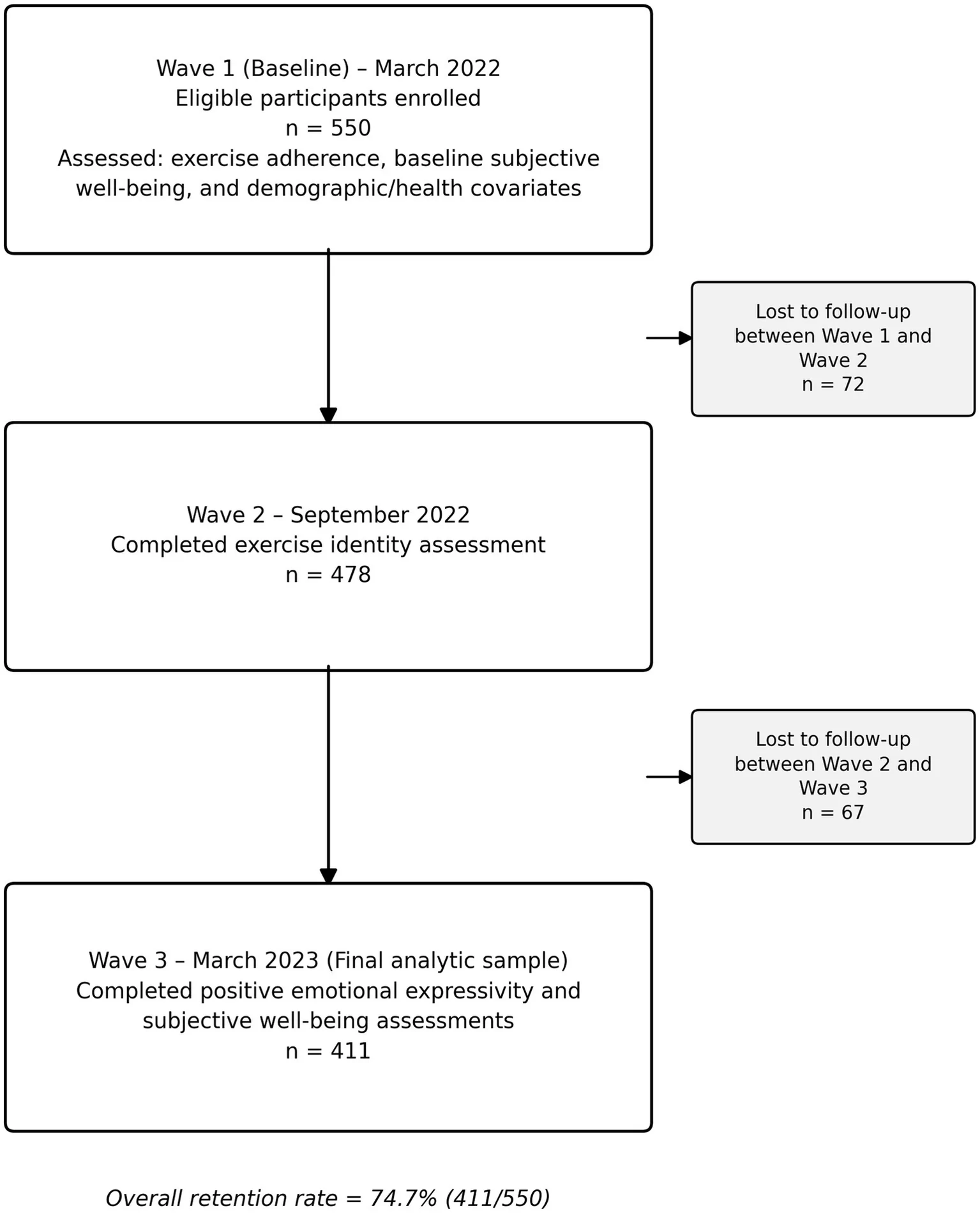
Participant flow across the three survey waves.

Data were collected at three time points separated by six-month intervals. At Wave 1 (baseline; March 2022), 550 eligible participants completed measures of exercise adherence, baseline subjective wellbeing, and demographic and health covariates. At Wave 2 (September 2022), 478 participants completed the exercise identity assessment. At Wave 3 (March 2023), 411 participants completed measures of positive emotional expressivity and subjective wellbeing. The overall retention rate was 74.7% (see Figure 2). Participants received a modest incentive after each completed survey wave and were provided with general health education materials. Baseline demographic characteristics and attrition analyses are presented in Table 1.

**Table 1:** Baseline demographic characteristics and attrition analysis.

Variable	Total sample (*N* = 550)	Retained sample (*n* = 411)	Attrited sample (*n* = 139)	Test statistic	*p*
Gender, *n* (%)				*χ*^2^ = 0.45	0.502
Male	240 (43.6%)	180 (43.8%)	60 (43.2%)		
Female	310 (56.4%)	231 (56.2%)	79 (56.8%)		
Education level, *n* (%)				*χ*^2^ = 0.82	0.664
High school or below	272 (49.5%)	200 (48.7%)	72 (51.8%)		
Associate/Bachelor’s degree	212 (38.5%)	161 (39.2%)	51 (36.7%)		
Graduate degree	66 (12.0%)	50 (12.1%)	16 (11.5%)		
Age, years, M (SD)	58.85 (6.90)	58.72 (6.81)	59.24 (7.18)	*t* = −0.77	0.442
Self-rated health, M (SD)	3.20 (0.76)	3.21 (0.78)	3.16 (0.72)	*t* = 0.66	0.510
T1 exercise adherence, M (SD)	3.22 (0.83)	3.24 (0.81)	3.15 (0.89)	*t* = 1.09	0.276
T1 subjective wellbeing, M (SD)	3.40 (0.75)	3.41 (0.76)	3.36 (0.71)	*t* = 0.68	0.497

Continuous variables were compared with independent-samples *t*-tests; categorical variables were compared with chi-square tests.

### 2.2 Measures

Because the original scales were developed in English, all measurement instruments were adapted into Chinese following a rigorous forward-backward translation procedure (Brislin, 1970). Content validity was evaluated by three experts in sports psychology and gerontology. Prior to the formal study, a pilot test was conducted with a separate sample of 45 community-dwelling older adults to evaluate item comprehension.

Exercise adherence. Exercise adherence was measured at baseline using a six-item self-report scale adapted from the Exercise Adherence Rating Scale (Newman-Beinart et al., 2017). All six items were reworded to replace references to clinical prescriptions with planned community-based exercise (e.g., changing “my prescribed exercises” to “my planned exercises”). The adapted Chinese items and their English counterparts are available from the corresponding author upon reasonable request. Participants rated the items on a 5-point Likert scale ranging from 1 (strongly disagree) to 5 (strongly agree). Item responses were averaged to create a scale score (possible range: 1–5), with higher scores indicating stronger adherence. This operationalization is consistent with behavior-maintenance perspectives (Kwasnicka et al., 2016). In the pilot test, the adapted Chinese version demonstrated good preliminary internal consistency (*α* = 0.85). Cronbach’s alpha in the present sample was 0.87.

Exercise identity. Exercise identity was assessed at Wave 2 using five items adapted from the Exercise Identity Scale (Anderson and Cychosz, 1994). Items assessed the salience of exercise as part of the self-concept. Participants responded on a 5-point Likert scale (1 = strongly disagree, 5 = strongly agree). No items were reverse-scored. Responses were averaged to yield a final score (possible range: 1–5). Cronbach’s alpha was 0.89 (see Table 2).

**Table 2:** Reliability and convergent validity of the measurement model.

Construct	Time point	Items	Loading range	Cronbach’s *α*	CR	AVE
Exercise adherence	T1	6	0.68–0.84	0.87	0.88	0.55
Subjective wellbeing	T1	7	0.65–0.81	0.88	0.89	0.53
Exercise identity	T2	5	0.72–0.88	0.89	0.89	0.61
Positive emotional expressivity	T3	4	0.70–0.85	0.86	0.87	0.63
Subjective wellbeing	T3	7	0.67–0.83	0.89	0.90	0.55

CR, composite reliability; AVE, average variance extracted. All standardized factor loadings were statistically significant at *p* < 0.001. For subjective wellbeing, Cronbach’s *α* was calculated using the 15 original items, whereas CR, AVE, and factor-loading ranges were based on the seven indicators used in the latent measurement model: five SWLS items and two PANAS positive-affect parcels.

Positive emotional expressivity. Positive emotional expressivity was assessed at Wave 3 using a context-adapted four-item measure based on the Berkeley Expressivity Questionnaire (Gross and John, 1997). Responses were given on a 5-point Likert scale (1 = strongly disagree, 5 = strongly agree) and averaged to form a composite score (possible range: 1–5). Cronbach’s alpha was 0.86.

Subjective wellbeing. Subjective wellbeing (SWB) was assessed at baseline and Wave 3 as a composite of cognitive life satisfaction and positive affect. Life satisfaction was measured with the five-item Satisfaction With Life Scale (Diener et al., 1985; Pavot and Diener, 1993), rated on a standard 7-point Likert scale (1 = strongly disagree, 7 = strongly agree). Positive affect was measured with the 10-item positive affect subscale of the Positive and Negative Affect Schedule (Watson et al., 1988), using the timeframe “during the past few weeks” and rated on a 5-point scale (1 = very slightly or not at all, 5 = extremely). For descriptive analyses (e.g., Table 3), the SWLS mean score was linearly rescaled to a 1–5 metric. The rescaled SWLS mean and the PANAS positive-affect mean were then averaged, giving equal weight to the cognitive and affective components of subjective wellbeing. However, for structural equation modeling and longitudinal measurement invariance testing (Table 4), SWB was modeled as a latent construct with seven indicators: the five individual SWLS items and two item parcels created from the 10 PANAS positive affect items using the item-to-construct balance technique (Little et al., 2002). This seven-indicator specification corresponds to the change of 6 degrees of freedom (representing 6 constrained factor loadings or intercepts) observed across the nested invariance models. Cronbach’s alpha values for the combined items were 0.88 at baseline and 0.89 at Wave 3.

**Table 3:** Descriptive statistics, correlations, and discriminant validity (*N* = 411).

Variable	M	SD	1	2	3	4	5
1. T1 exercise adherence	3.24	0.81	(0.74)				
2. T1 subjective wellbeing	3.41	0.76	0.28***	(0.73)			
3. T2 exercise identity	3.12	0.85	0.36***	0.32***	(0.78)		
4. T3 positive emotional expressivity	3.35	0.79	0.29***	0.35***	0.41***	(0.79)	
5. T3 subjective wellbeing	3.52	0.83	0.32***	0.42***	0.35***	0.38***	(0.74)

Diagonal values in parentheses are square roots of AVE. ***p < 0.001.

**Table 4:** Longitudinal measurement invariance of subjective wellbeing.

Model	*χ* ^2^	df	CFI	TLI	RMSEA [90% CI]	SRMR	Comparison	ΔCFI	ΔRMSEA
M1 configural	213.45	122	0.972	0.965	0.043 [0.032, 0.054]	0.038	—	—	—
M2 metric	221.38	128	0.969	0.963	0.042 [0.031, 0.053]	0.040	M2 vs. M1	0.003	0.001
M3 scalar	229.72	134	0.964	0.960	0.041 [0.030, 0.052]	0.042	M3 vs. M2	0.005	0.001

Invariance was evaluated using changes in CFI and RMSEA, following commonly used recommendations for measurement invariance testing.

Covariates. Baseline covariates included age, gender, educational attainment, and self-rated physical health. These covariates were selected because demographic and health-related variables are commonly associated with both physical activity and wellbeing in aging populations (McAuley et al., 2006; Steptoe et al., 2015). Self-rated health was included because perceived health may influence both the ability to maintain exercise and overall life evaluation. Education was included as a broad indicator of socioeconomic resources that may affect access to exercise opportunities and health information. For statistical modeling, baseline covariates were coded and scored as follows: gender was dummy-coded (0 = male, 1 = female); educational attainment was coded as an ordinal variable (1 = high school or below, 2 = Associate/Bachelor’s degree, 3 = Graduate degree); and self-rated physical health was scored on a 5-point Likert scale ranging from 1 (poor) to 5 (excellent). Age was treated as a continuous variable measured in years.

### 2.3 Data analysis

Data analyses were conducted using IBM SPSS Statistics 26.0 and Mplus 8.3 (Muthén and Muthén, 1998–2017). Descriptive statistics, correlations, attrition analyses, and initial assumption checks were conducted in SPSS. Structural equation modeling was conducted in Mplus. Missing item-level data among completers were handled using full information maximum likelihood, which is commonly recommended for longitudinal models when data are plausibly missing at random (Little, 2013). Statistical significance of indirect effects was evaluated with bias-corrected bootstrapping based on 5,000 resamples, following contemporary mediation-analysis recommendations (Hayes, 2018).

The analytic strategy proceeded in four steps. First, attrition analyses compared retained and attrited participants on baseline variables. Second, confirmatory factor analysis was used to evaluate the measurement model. Reliability was evaluated using Cronbach’s alpha and composite reliability, convergent validity using average variance extracted, and discriminant validity by comparing the square root of AVE with inter-construct correlations (Fornell and Larcker, 1981). Third, longitudinal measurement invariance was tested only for subjective wellbeing, because it was the only focal latent construct measured at both baseline and 12 months. Configural, metric, and scalar invariance models were tested sequentially. The metric invariance model constrained factor loadings to be equal across the two waves, whereas the scalar invariance model additionally constrained item intercepts to be equal. Following commonly used recommendations, measurement invariance was considered supported when the decrease in CFI did not exceed 0.010 (Cheung and Rensvold, 2002) and the increase in RMSEA did not exceed 0.015 (Chen, 2007) between successive models. Fourth, the hypothesized mediation model was tested while controlling for baseline subjective wellbeing and covariates. Model fit was evaluated using *χ*^2^/df, CFI, TLI, RMSEA, and SRMR, with interpretation guided by commonly used SEM recommendations (Hu and Bentler, 1999; Kline, 2016).

## 3 Results

### 3.1 Preliminary analyses and measurement model

All continuous variables showed acceptable distributional properties, with absolute skewness values ranging from 0.12 to 0.85 and kurtosis values ranging from 0.22 to 1.14. Harman’s single-factor test suggested that the first unrotated factor accounted for 28.7% of the total variance; however, given the known limitations of this diagnostic and the broader concerns surrounding common method variance in self-report research (Podsakoff et al., 2003), common method bias is considered a limitation rather than ruled out. The confirmatory measurement model showed acceptable reliability and convergent validity. Standardized factor loadings ranged from 0.65 to 0.88, composite reliability ranged from 0.87 to 0.90, and AVE ranged from 0.53 to 0.63 (see Figure 1).

### 3.2 Longitudinal measurement invariance of subjective wellbeing

Because subjective wellbeing was assessed at both baseline and 12 months, longitudinal measurement invariance was tested for this construct. The configural model showed acceptable fit. Imposing equality constraints on factor loadings and intercepts did not meaningfully reduce fit, supporting the use of the subjective wellbeing construct across the two waves. This treatment avoids overstating invariance across variables that were measured at only one wave and follows the principle that longitudinal comparisons require evidence that the repeated construct retains comparable measurement meaning over time (Little, 2013).

### 3.3 Structural equation modeling and mediation

The hypothesized structural model showed good fit to the data: $\chi^{2}$ = 400.68, df = 212, $\chi^{2}$ /df = 1.89, CFI = 0.967, TLI = 0.960, RMSEA = 0.045, 90% CI [0.030, 0.058], SRMR = 0.041. The total effect of baseline exercise adherence on 12-month subjective wellbeing was significant ( β=0.240 , SE = 0.042, z=5.71 , 95% CI [0.160, 0.320], *p* < 0.001). After exercise identity and positive emotional expressivity were included, the direct effect was attenuated and became non-significant ( β=0.079 , SE = 0.051, z=1.55 , 95% CI [−0.021, 0.179], *p* = 0.126).

**Figure 3:**
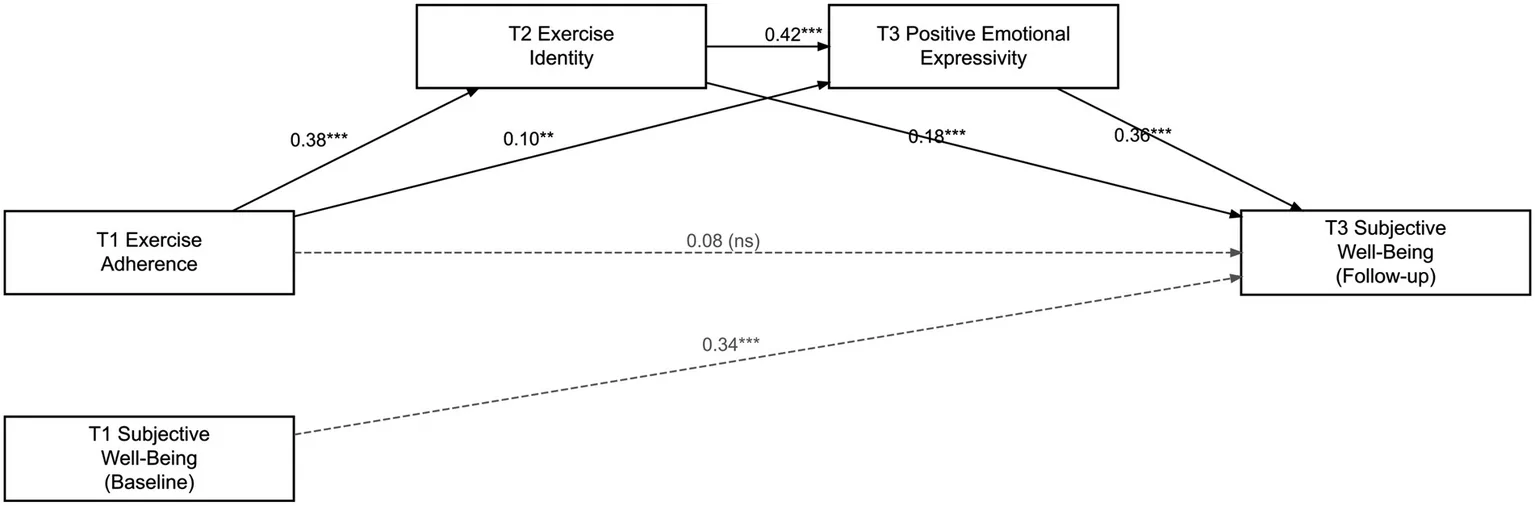
Standardized structural model linking exercise adherence, exercise identity, positive emotional expressivity, and subjective wellbeing. * *p* < 0.05, ** *p* < 0.01, *** *p* < 0.001.

Baseline exercise adherence positively predicted exercise identity at 6 months ( β=0.380 , SE = 0.053, z=7.17 , *p* < 0.001). Because all structural coefficients were standardized, a coefficient of β=0.380 indicates that a one-standard-deviation increase in baseline exercise adherence was associated with a 0.380-standard-deviation increase in exercise identity at 6 months. Exercise identity positively predicted positive emotional expressivity at 12 months ( β=0.420 , SE = 0.051, z=8.24 , *p* < 0.001), and positive emotional expressivity positively predicted subjective wellbeing at 12 months ( β=0.360 , SE = 0.052, z=6.92 , *p* < 0.001). Figure 3 presents the individual standardized path coefficients rounded to two decimal places. To address the necessity of reporting comprehensive statistical tests, Table 5 presents the complete structural path estimates—including all direct paths, indirect paths, and control variable paths—along with their corresponding Mplus z -statistics (*Est./S.E.*).

**Table 5:** Comprehensive standardized structural path estimates, including direct, indirect, and covariate effects.

Path/effect	Estimate ( β )	SE	z *	*p*	Bootstrap 95% CI
Structural paths (direct)					
T1 adherence → T2 identity	0.380	0.053	7.17	<0.001	[0.276, 0.484]
T1 adherence → T3 expressivity	0.100	0.050	2.00	0.045	[0.002, 0.198]
T1 adherence → T3 SWB	0.079	0.051	1.55	0.126	[−0.021, 0.179]
T2 identity → T3 expressivity	0.420	0.051	8.24	<0.001	[0.320, 0.520]
T2 identity → T3 SWB	0.179	0.052	3.44	0.001	[0.077, 0.281]
T3 expressivity → T3 SWB	0.360	0.052	6.92	<0.001	[0.258, 0.462]
T1 SWB (baseline) → T3 SWB	0.340	0.052	6.54	<0.001	[0.238, 0.442]
Covariate paths (on T3 SWB)					
Age → T3 SWB	0.042	0.045	0.93	0.352	[−0.046, 0.130]
Gender (female = 1) → T3 SWB	0.065	0.043	1.51	0.131	[−0.019, 0.149]
Education → T3 SWB	0.112	0.046	2.43	0.015	[0.022, 0.202]
T1 self-rated health → T3 SWB	0.154	0.047	3.28	0.001	[0.062, 0.246]
Mediation effects (indirect)					
Ind 1: adherence → identity → SWB	0.068	0.020	3.40	<0.001	[0.029, 0.107]
Ind 2: adherence → expressivity → SWB	0.036	0.010	3.60	<0.001	[0.016, 0.056]
Ind 3: adherence → identity → expressivity → SWB	0.057	0.017	3.35	<0.001	[0.028, 0.094]
Total indirect effect	0.161	0.030	5.37	<0.001	[0.102, 0.220]
Total effect (adherence → T3 SWB)	0.240	0.042	5.71	<0.001	[0.160, 0.320]

SWB, subjective wellbeing. ^*^The z -statistic represents the Est./S.E. ratio calculated via Mplus maximum likelihood estimation. Control variables were modeled to predict the final outcome variable (T3 SWB). * *p* < 0.05, ** *p* < 0.01, *** *p* < 0.001.

## 4 Discussion

### 4.1 Principal findings

This three-wave longitudinal study examined psychological pathways linking exercise adherence with subjective wellbeing among middle-aged and older adults. Consistent with the hypotheses, baseline exercise adherence was prospectively associated with higher subjective wellbeing at 12 months. This association was statistically explained by exercise identity and positive emotional expressivity, including a significant sequential indirect pathway. These findings are consistent with broader evidence that physical activity is associated with wellbeing in adulthood and later life (Buecker et al., 2021; Netz et al., 2005), but they extend that evidence by focusing on sustained adherence and by examining psychological mechanisms that may translate repeated exercise behavior into subjective wellbeing.

The association between exercise adherence and later exercise identity supports the idea that repeated behavioral enactment can strengthen a corresponding role identity. For aging adults, consistent exercise may provide evidence that one is a capable, active, and health-oriented person. Such an identity may be especially meaningful during midlife and later-life transitions when previous work and family roles may shift. The present findings are consistent with identity theory (Stryker, 1968; Stryker and Burke, 2000), with the Exercise Identity Scale tradition (Anderson and Cychosz, 1994), and with meta-analytic evidence linking physical activity identity to physical activity participation (Rhodes et al., 2016). They also accord with self-determination theory in suggesting that exercise may be more sustainable when it is internalized and experienced as congruent with the self rather than merely imposed by external health advice (Deci and Ryan, 2000).

The results also highlight the affective dimension of exercise participation. Exercise identity was associated with positive emotional expressivity, which in turn was related to subjective wellbeing. This finding is consistent with broaden-and-build theory and with affective perspectives on health behavior change (Fredrickson, 2001, 2004; van Cappellen et al., 2018). Openly expressing positive feelings in exercise settings may help older adults consolidate feelings of accomplishment, strengthen social bonds, and increase the emotional rewards associated with physical activity. These processes may contribute to broader evaluations of life satisfaction and positive affect, especially because emotional meaning and social connectedness are important aspects of adaptation in later adulthood (Carstensen et al., 1999; Charles and Carstensen, 2010).

The finding that the direct path from exercise adherence to follow-up subjective wellbeing became non-significant after the mediators were included should not be interpreted as evidence that physiological mechanisms are irrelevant. Exercise can influence wellbeing through multiple pathways, including physical functioning, self-efficacy, social participation, sleep, and disease management (Chodzko-Zajko et al., 2009; McAuley et al., 2006; Rejeski and Mihalko, 2001). The present model isolates a psychological pathway and shows that identity and positive emotional expression are statistically important in this dataset. Future studies could compare psychological, social, and physiological mediators within the same longitudinal model.

### 4.2 Theoretical contributions

The study contributes to the literature in three ways. First, it integrates identity theory with the broaden-and-build theory of positive emotions to explain how sustained exercise behavior may become psychologically meaningful. This integration links a cognitive self-concept mechanism with an affective and social mechanism, thereby providing a more complete account of how exercise adherence may be associated with wellbeing. Second, the three-wave design helps establish temporal ordering between exercise adherence and exercise identity and reduces some limitations of cross-sectional mediation models. Third, the study focuses on adherence rather than exercise frequency alone, emphasizing the sustained behavioral maintenance that is particularly relevant for healthy aging and for the design of public health programs (Bull et al., 2020; Kwasnicka et al., 2016).

At the same time, the results should be understood as evidence of longitudinal associations and statistical mediation, not as definitive proof of causal mechanisms. Stronger causal inference would require randomized interventions, objective behavioral measures, and additional follow-up waves that temporally separate all mediators and outcomes.

Methodologically, the study also illustrates a cautious approach to longitudinal mediation. Measurement invariance was examined only for the construct measured at multiple waves, and the mediation interpretation was explicitly limited because the final mediator and outcome were measured concurrently. This is important because excellent model fit does not guarantee causal validity, and SEM results should be interpreted in light of design features, measurement limitations, and theoretical plausibility (Kline, 2016; Little, 2013).

### 4.3 Practical implications

The findings have implications for community exercise programs for middle-aged and older adults. Programs may be more effective when they go beyond monitoring attendance or exercise volume and deliberately foster exercise-related identity. Possible strategies include stable group membership, peer recognition, participant roles such as warm-up leader or activity recorder, and progress feedback that helps participants see themselves as regular exercisers. These practices are compatible with the idea that repeated participation can strengthen role identity and with public health guidance emphasizing regular, sustainable activity rather than short-term effort alone (Rhodes et al., 2016; World Health Organization, 2020).

Programs may also benefit from creating emotionally supportive exercise environments. Instructors can encourage participants to share positive experiences, acknowledge personal progress, and provide supportive feedback to peers. For older adults with limited mobility, low-intensity group activities may still provide psychological benefits if they promote social connection, mastery, and positive affective expression. Such design features are consistent with research on positive affective processes in health behavior change and with evidence that later-life emotional goals often prioritize meaningful and supportive social experiences (Carstensen et al., 1999; van Cappellen et al., 2018).

### 4.4 Limitations and future research

Several limitations should be noted. First, the study was observational. Although baseline subjective wellbeing and covariates were controlled, unmeasured time-varying factors such as changes in health, chronic disease burden, social support, family stress, socioeconomic status, exercise motivation, neighborhood resources, sleep quality, or medication use may have influenced both exercise and wellbeing. Therefore, causal interpretations should be cautious. Randomized controlled trials, natural experiments, or intervention designs that manipulate program features related to identity and emotional expression would provide stronger causal evidence.

Second, positive emotional expressivity and follow-up subjective wellbeing were both measured at Wave 3. This means the final link in the proposed chain does not have full temporal separation. Future studies should use four or more waves, such as measuring adherence at T1, identity at T2, positive emotional expressivity at T3, and subjective wellbeing at T4. Cross-lagged or latent growth models could also be used to examine whether changes in identity and emotion expression predict subsequent changes in wellbeing over time (Little, 2013).

Third, all focal variables were assessed by self-report. Although the longitudinal design reduces some single-wave method concerns, self-report exercise adherence is vulnerable to recall bias and social desirability, and emotional expressivity may be influenced by personality, cultural norms, and response tendencies. The use of the same assessment method across the focal variables may have inflated the observed associations and indirect effects through shared response tendencies. Future research should combine self-report scales with objective measures such as accelerometers, activity logs, attendance records, ecological momentary assessment, or instructor-rated adherence and expressive behavior. More sophisticated approaches to common method bias, such as latent method-factor models or marker variables, would also strengthen future research (Podsakoff et al., 2003).

Fourth, the sample was recruited from urban community settings in one Chinese province. Participants likely had relatively good access to exercise resources, community programs, and health information. Moreover, individuals attending community centers and senior universities may be healthier, more physically active, and more socially engaged than the general middle-aged and older population, which may have introduced selection bias and further limited the generalizability of the findings. Generalizability to rural populations, clinical populations, adults with severe mobility limitations, or culturally different settings requires further testing. Because social exercise norms and emotional display rules differ across cultures, the relationship between exercise identity, positive emotional expressivity, and wellbeing may vary across social contexts.

Finally, the study did not include several theoretically relevant variables, such as social support, exercise enjoyment, autonomous motivation, perceived competence, depressive symptoms, negative affect, or objective health status. Including these variables would allow future studies to test competing or complementary mechanisms. For example, self-determination theory would suggest that autonomy and competence may explain why identity becomes internalized (Deci and Ryan, 2000), whereas subjective wellbeing theory would encourage separate examination of life satisfaction, positive affect, and negative affect rather than relying only on a composite score (Diener et al., 2018; Watson et al., 1988).

## 5 Conclusion

This study provides longitudinal evidence that exercise adherence is associated with later subjective wellbeing among middle-aged and older adults and that this association is statistically explained by exercise identity and positive emotional expressivity. Sustained exercise may enhance wellbeing by helping individuals internalize an exerciser identity and express positive emotions in exercise-related contexts. Community exercise programs should therefore attend to psychological identity formation and positive affective experiences alongside physical activity targets. Given the observational design and the concurrent measurement of the final mediator and outcome, these findings should be interpreted as supportive but not conclusive evidence of the proposed psychological pathway. Future intervention studies with objective adherence measures and temporally separated mediators and outcomes are needed to determine whether deliberately cultivating exercise identity and positive emotional expression can produce durable improvements in wellbeing.

## Data Availability

The raw data supporting the conclusions of this article will be made available by the authors, without undue reservation.
